# Clinical management and long-term outcomes in pulmonary inflammatory myofibroblastic tumor: a 12-Year experience with 14 surgically treated adult patients at a single center

**DOI:** 10.1186/s12957-025-03973-1

**Published:** 2025-08-28

**Authors:** Wenhao Wang, Xuan Wang, Haoxin Liu, Dong Xu, Kaiheng Gao, Yulong Tan, Zhouyi Lu, Wan Posum, Meng Shi, Huijun Zhang, Xiaofeng Chen

**Affiliations:** Department of Thoracic Surgery, Huashan Hospital & Cancer Metastasis Institute, Fudan University, Shanghai, 200040 China

**Keywords:** Inflammatory myofibroblastic tumor, VATS, Anaplastic lymphoma kinase, Prognosis

## Abstract

**Background:**

Pulmonary inflammatory myofibroblastic tumor (PIMT) is a rare, borderline mesenchymal neoplasm with unclear etiology. It carries recurrence risks but lacks robust data on surgical outcomes in adults. This study analyzes clinicopathological features and long-term results of surgically managed adult PIMT patients at a single center.

**Methods:**

A retrospective analysis of 14 adults (9 male, 5 female; mean age 47.6± 14.1 years) undergoing surgical resection for pathologically confirmed PIMT (2012-2023) at a single institution. All patients underwent video-assisted thoracoscopic surgery. Immunohistochemistry was systematically analyzed. The median follow-up for all patients was 53 months (range, 24-122 months).

**Results:**

Common presenting symptoms included cough (35.7%) , chest tightness (28.6%), and asymptomatic (28.6%). Mean tumor diameter was 3.1± 0.9 cm. Complete (R0) resection was achieved in all cases. No recurrence or metastasis was observed to the time of writing.

**Conclusion:**

PIMT is a rare tumor requiring pathological confirmation. Complete surgical resection (R0) via video-assisted thoracoscopic surgery is the preferred curative approach, demonstrating excellent long-term outcomes in this cohort with no recurrence or metastasis observed, but close surveillance is essential due to potential recurrence risk.

## Background

Inflammatory myofibroblastic tumor (IMT) is a rare mesenchymal neoplasm. IMTs occur primarily in the abdominal cavity (75% of cases) and have also been reported in orbit, head and neck, heart, lung, esophagus, extremities, abdomen, bladder and genitourinary system [1–9]. Pulmonary inflammatory myofibroblastic tumor (PIMT) is even more uncommon, accounting for only 0.04–0.7% of all lung masses [10]. The etiology of PIMT remains unclear, and current research predominantly consists of small-sample studies or case reports [11]. PIMT can occur at any age but is more frequently documented in children [12]. Clinical summaries focusing on adult PIMT, particularly regarding surgical outcomes and prognosis remain scarce. Currently, PIMT is considered as a borderline tumor pathologically characterized by myofibroblastic proliferation and varying degrees of inflammatory cell infiltration, with potential risks of recurrence and metastasis [13]. Due to its rarity, clinicians possess limited understanding of its clinical manifestations, pathological features, surgical efficacy, and prognosis. This study seeks to summarize the surgical outcomes, clinical management experience, and long-term prognosis of 14 adult PIMT patients treated surgically at a single center over a 10-year period, aiming to offer valuable insights for clinical practice.

## Methods

### Study design and patient information

This single-center, observational, retrospective cohort study was approved by the Huashan Hospital Ethics Committee (permit numbers: KY2019-434). Written informed consent was obtained from all participants in accordance with the Declaration of Helsinki (2013 revision). Patients pathologically diagnosed with PIMT following lung resection at Huashan Hospital, Fudan University, between January 2012 and May 2023 were identified. The inclusion criteria were patients who underwent thoracic surgery after assessment by the department of thoracic surgery at Huashan Hospital and were pathologically confirmed as PIMT. The exclusion criteria were patients with concurrent diagnosis of other malignancies and those with insufficient follow-up data (unknown clinicopathological characteristics, undocumented treatment, or missing follow-up). Cases were identified through the hospital’s electronic medical records and pathology database. Patient demographics, clinical characteristics, and outcomes were retrospectively reviewed. This study aimed to evaluate clinicopathological features and surgical treatment efficacy.

### Imaging evaluation

Preoperative investigations included laboratory tests and computed tomography (CT). For patients who did not undergo PET-CT, distant metastasis was ruled out using cranial magnetic resonance imaging (MRI), bone scintigraphy and abdominal ultrasonography, with no abnormalities detected in these assessments.

### Histopathology and immunohistochemistry

Surgical specimens were fixed in 10% neutral buffered formalin at room temperature for 24 h, dehydrated and equilibrated in ethanol, routinely embedded in paraffin, sectioned (4-µm thickness), and stained with hematoxylin and eosin (HE) at room temperature for 30 min, followed by immunohistochemical analysis.

### Treatment strategy

Management followed the NCCN Clinical Practice Guidelines in Oncology for Soft Tissue Sarcoma. Radical surgical resection via video-assisted thoracoscopic surgery (VATS) served as the primary treatment. No adjuvant therapy was administered after complete resection. In the event of recurrence or metastasis, subsequent management will be planned via multidisciplinary team discussion.

## Results

**Fig. 1:**
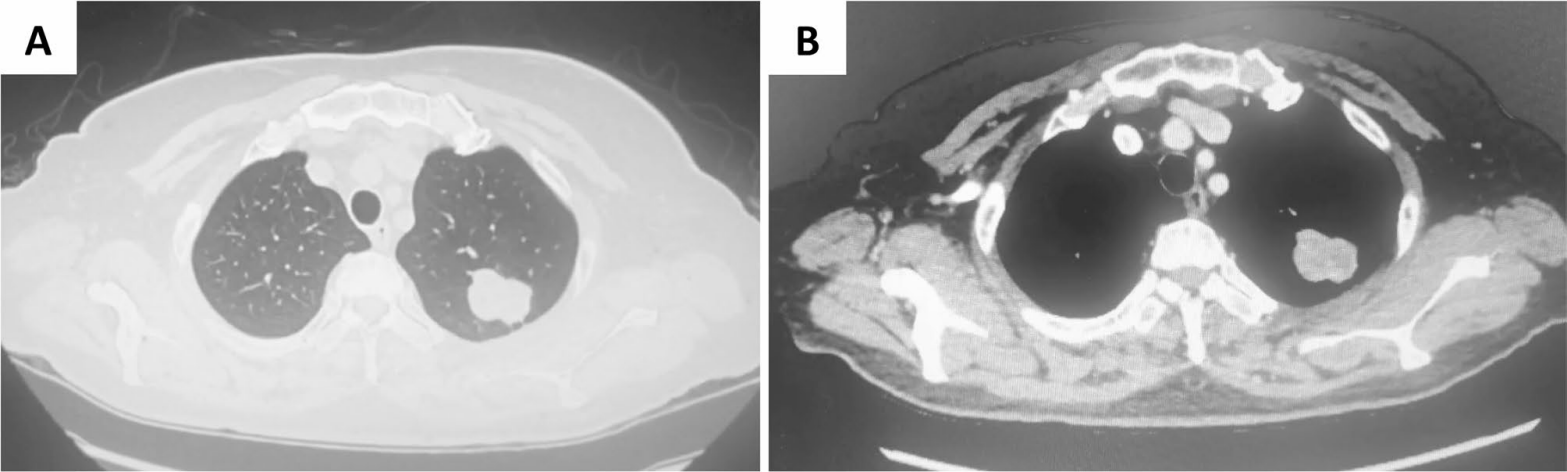
The CT scans of an inflammatory myofibroblastic tumor. **A** Lung window of CT shows a tumor measuring 3.2 cm in diameter in the apicoposterior segment of the left upper lobe, demonstrating a lobulated contour with peripheral spiculated margins. **B** Mediastinal window of chest CT shows no abnormal masses identified within the mediastinum. Both hila appear normal and pleural effusions are absent. Heterogeneous mild enhancement of the tumor is noted following contrast administration

**Fig. 2:**
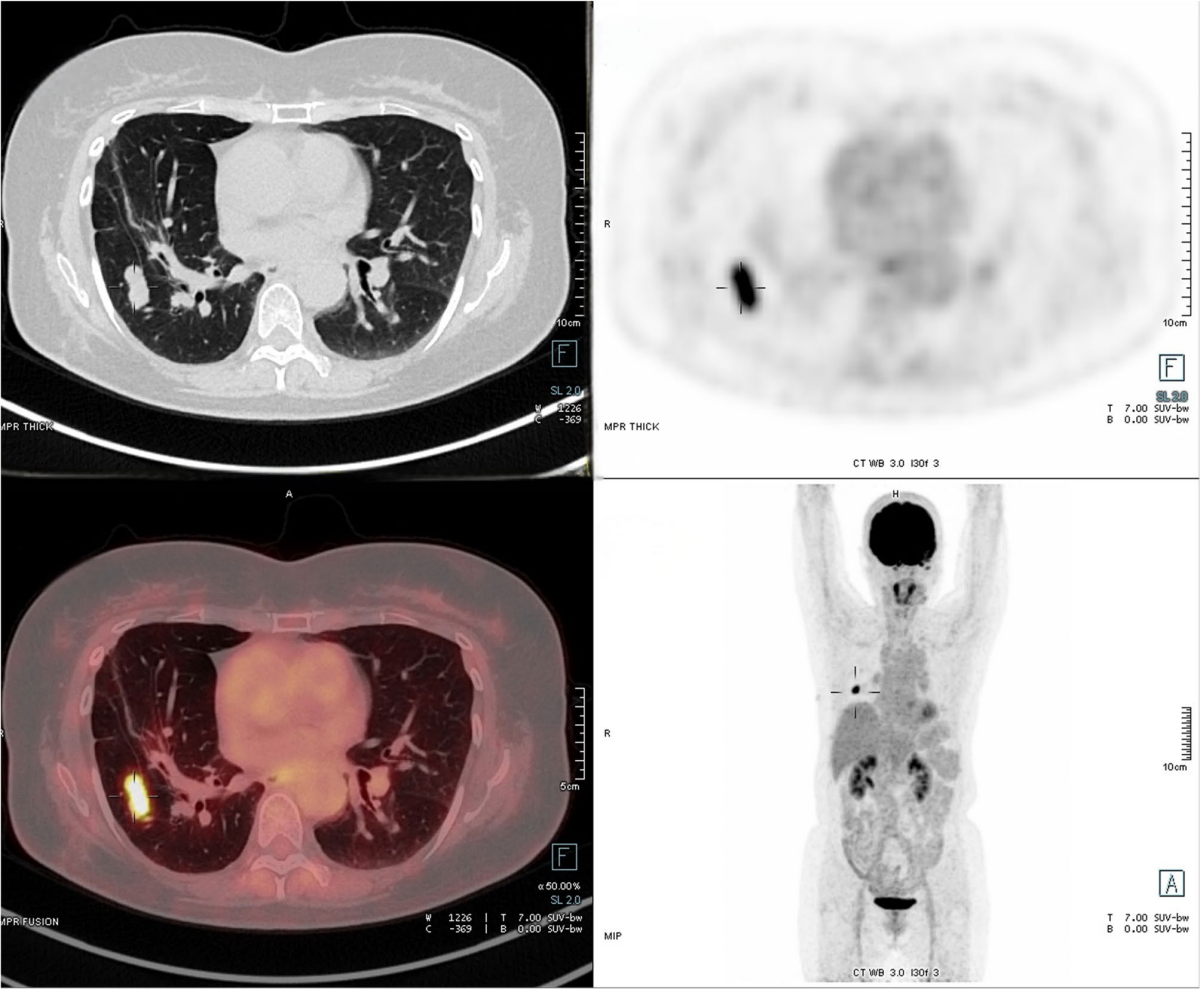
18F-FDG PET/CT revealed an irregular nodular opacity in the right lower lobe with relatively well-defined and smooth margins. The maximum axial dimension measured 2.8 × 1.2 cm, demonstrating intense FDG uptake (SUVmax = 11.4)

The clinical and pathological features of 14 patients are presented in Table 1. The cohort consisted of 9 male and 5 female, with ages ranging from 20 to 74 years (mean, 47.6 ± 14.1 years).Respiratory symptoms were present in 10 patients (71.4%), including cough (5 patients, 35.7%), chest tightness (4 patients, 28.6%), and hemoptysis (1 patient, 7.1%).Three patients (21.4%) were detected during physical examination, and 1 patient (7.1%) was found due to chest trauma. Tumor locations were: right upper lobe (2 patients, 14.3%), right middle lobe (3 patients, 21.4%), right lower lobe (4 patients, 28.6%), left upper lobe (4 patients, 28.6%), and left lower lobe (1 patient, 7.1%). Chest CT showed a median tumor diameter of 1.1–4.6 cm, with a maximum diameter of 3.1 ± 0.9 cm (Fig. 1). Six patients underwent preoperative PET - CT, with an average FDG uptake SUVmax of 9.6 ± 2.7 (range, 5.6 to 26.5). 13 patients had no history of thoracic surgery. Notably, patient eight had previously undergone VATS right pulmonary S1a combined S6b subsegmentectomy, with postoperative pathology showing minimally invasive adenocarcinoma (MIA). Six years postoperatively, a 2.9 cm mass was detected in the right lower lobe (Fig. 2). Subsequent VATS wedge resection of the right lower lobe pathology indicated PIMT.

**Table 1:** Patient characteristics and immunohistochemical labeling of operated inflammatory myofibroblastic tumors

Case	Age (years)/Sex	Symptoms	Localization/Size(cm)	Surgery	Immunohistochemical findings				Follow-up (month)
					ALK	SMA	CD34	Ki67	
1	48/M	Persistent cough	RLL/3.1	Lobectomy	+	+	-	10%	122
2	34/M	Chest tightness	LUL/3.4	Lobectomy	+	-	-	10%	109
3	20/F	Persistent cough	RML/1.1	Segmentectomy	+	+	+	15%	103
4	48/M	Asymptomatic	RML/1.9	Lobectomy	-	+	+	-	90
5	34/M	Chest tightness	RUL/3.7	Bronchial sleeve resection	+	+	-	10%	72
6	62/F	Persistent cough	RLL/4.1	Lobectomy	+	+	+	10%	64
7	55/M	Persistent cough	LLL/4.6	Lobectomy	+	-	+	5%	56
8	34/M	Chest tightness	RLL/2.9	Wedge	+	+	-	-	50
9	74/F	Trauma-associated	RLL/3.1	Wedge	+	+	-	5%	45
10	51/M	Persistent cough	RML/2.5	Lobectomy	+	+	+	5%	41
11	57/M	Asymptomatic	LUL/3.4	Lobectomy	-	+	-	10%	36
12	43/M	Hemoptysis	LUL/3.7	Bronchial sleeve resection	-	+	+	-	34
13	54/F	Chest tightness	LUL/3.2	Lobectomy	+	+	-	5%	28
14	56/F	Asymptomatic	RUL/3.3	Lobectomy	+	+	+	10%	24

*ALK* Anaplastic lymphoma kinase, *SMA* Smooth muscle actin, *IHC* Immunohistochemistry + positive, - negative, *percentage* Ki67 labeling index

All patients underwent VATS, including 11 lobectomies (78.6%), two wedge resections (14.3%), and one segmentectomy (7.1%). Two patients (14.3%) underwent bronchial sleeve resection due to tumor location at the bronchial orifices of the left upper lobe and right upper lobe, respectively. Complete tumor resection with negative margins was achieved in all patients. Intraoperative frozen sections suggested PIMT in 9 patients (64.3%), and no further lymph node dissection was performed. However, lymph node dissection was conducted in 5 patients (35.7%) due to inconclusive frozen section results. None developed postoperative complications and the mean hospital stay was 4.3 ± 1.2 days (range, 2–6 days), with a mean postoperative drainage volume of 415 ± 134 mL (range, 170–730 mL).

**Fig. 3:**
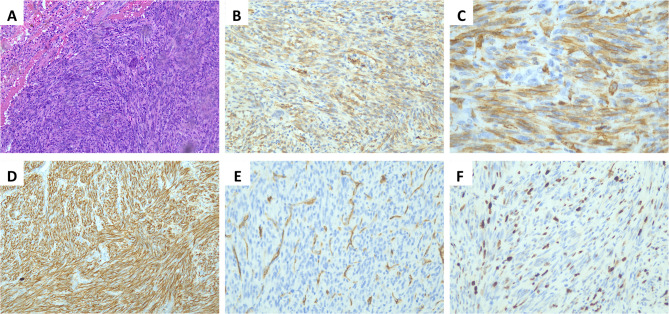
Histological features of PIMT. **A** Spindle-shaped tumor cells surrounded by chronic inflammatory infiltrate with HE staining at 100× magnification. **B** ALK positivity with IHC staining at 200× magnification **C** ALK positivity with IHC staining at 400× magnification. **D** SMA positivity with IHC staining at 200× magnification. **E** CD34 positivity with IHC staining at 200× magnification. **F** Ki67 positivity with IHC staining at 200× magnification

All pathological sections were reviewed and confirmed by two pathologists with at least 10 years’ experience at our institution. Histologically, tumors consisted predominantly of spindle cells surrounded by chronic inflammatory cell infiltration (Fig. 3A). Immunohistochemical analysis revealed anaplastic lymphoma kinase (ALK) positivity in 10 patients (71.4%, Fig. 3B, C), smooth muscle actin (SMA) positivity in 12 patients (85.7%, Fig. 3D), CD34 positivity in 7 patients (50%, Fig. 3E) and Ki67 positivity in 11 patients (78.6%, Fig. 3F). Based on pathological features and immunohistochemical profiles, all 14 patients were diagnosed as PIMT. Lymph nodes were negative in all 5 patients who underwent lymph node dissection.

All patients received no further treatment after discharge. Follow-up was based on clinical evaluation, with chest CT scans performed every six months for the first three years and annually thereafter. The median follow-up was 53 months (range, 24–122 months). To date, all patients remain alive with no tumor recurrence or distant metastasis detected.

## Discussion

PIMT represents the most common mesenchymal lung tumor in individuals under 16 years, yet accounts for less than 1% of all lung tumors [14]. Previous studies predominantly indicate no significant influence of gender on PIMT occurrence [15]. Three prior Korean studies reported male proportions of 67%, 82%, and 62.5%, respectively among PIMT patients [16–18]. Our study revealed a male proportion of 57.1%, a variation potentially attributable to sampling differences across case series. This study provides further supplementation to the epidemiological data for this disease. Some studies suggest an endobronchial location may be a relatively common site for PIMT [18]while a retrospective study including 8 PIMT patients showed 75% occurred in the right lung [19]. Similarly, our study found a higher incidence in the right lung (64.3%) compared to the left lung(35.7%), consistent with the aforementioned result.

The etiology and pathogenesis of PIMTs remain unclear. Notably, patient eight had pre-existing MIA and developed a new ipsilateral PIMT. Histopathological analysis excluded recurrence or metastasis, suggesting a second primary tumor. This may imply independent oncogenic pathways for adenocarcinoma and PIMT, warranting further genetic analysis. To our knowledge, this is the first report of PIMT developing after lung adenocarcinoma resection. To date, no direct evidence suggests an association between PIMT occurrence and previous pulmonary surgery. The 50-month disease-free survival (DFS) post PIMT resection in this case reinforces its distinct biological behavior.

The clinical symptoms of PIMT are non-specific, primarily including cough, hemoptysis, chest pain, and dyspnea. Approximately 30% of patients may present with systemic symptoms such as fever and weight loss [20, 21]. In our study, four patients (28.6%) exhibited cough as the predominant symptom, which may be associated with local tumor invasion and inflammation. Four patients were asymptomatic, and one case was incidentally discovered following chest trauma, further illustrating the non-specific nature of PIMT symptoms.

Preoperative definitive diagnosis of PIMT is challenging. Differentiating PIMT from lung cancer, carcinoid tumors, and specific pulmonary infections such as tuberculosis, aspergillosis, cryptococcosis, and hamartoma can be difficult [22]. Therefore, routine examinations are essential prior to pulmonary surgery. CT and PET-CT are commonly employed auxiliary examinations for PIMT, though their findings may also lack specificity [23]. CT is utilized in nearly all PIMT cases, typically revealing a solitary pulmonary nodule of variable morphology. Some may appear as round, high-density nodules without lymphadenopathy [24]. Contrast-enhanced CT predominantly demonstrates varying degrees of delayed enhancement. The density may appear heterogeneous or exhibit central low-density necrosis. Mild delayed enhancement may be observed upon contrast administration, potentially related to abundant tumor vascularity accompanied by partial inflammation [25].The role of FDG PET-CT in IMT is limited. A previous study including 7 IMT patients and one spindle cell sarcoma patient transformed from IMT reported a mean SUVmax of 10.9 ± 5.5 (range, 3.3 to 20.8) [26]. Its utility is confined to monitoring local recurrence, treatment response, and distant metastasis. Another study has found diagnostic value of PET-CT in IMT in cases associated with other systemic diseases, such as G-CSF-producing inflammatory myofibroblastic tumor [27]. In our series, 6 patients underwent preoperative 18F-FDG PET/CT, revealing variable FDG uptake ranging from low to high intensity, with a mean SUVmax of 9.6 ± 2.7 (range, 5.6 to 26.5). This variability may be related to tumor density, biological behavior, and the activation level of inflammatory cells. It is critical to note that high SUVmax values may mimic malignancy, necessitating pathological confirmation to avoid overtreatment.

Although rare cases of spontaneous regression [28] of PIMT or cure following short-term hormonal therapy [29], surgical resection remains the preferred approach for PIMT. Frozen section examination is crucial for establishing a diagnosis intraoperatively and determining the extent of resection, including the decision for lymph node dissection. However, intraoperative frozen section diagnosis of PIMT is also challenging [16, 30]. Yüksel C et al. [31] performed frozen section analysis on 10 surgical patients ultimately confirmed as PIMT by final pathology, only 2 patients (20%) were diagnosed as PIMT intraoperatively. In contrast, in our case series, frozen sections suggested PIMT in nine patients (64.3%), reflecting greater expertise among our pathologists in identifying these tumors. Considering reports of PIMT recurrence characteristics in some studies, our approach is to perform a moderately wider resection when feasible and tolerated by the patient. No lymph node metastasis occurred in 5 patients undergoing dissection, reinforcing that routine lymphadenectomy is unwarranted. We recommend selective dissection only when malignancy cannot be excluded intraoperatively, consistent with the findings of Thistlethwaite et al. [32]. Data regarding survival rates in PIMT are limited in the literature. Studies by Zhu (eight patients) [19], Jeong (eight patients) [18]and Lee(15 patients) [16] reported no mortalities, with a 100% overall survival (OS) rate, which aligns with our 100% OS outcome. Importantly, our cohort also demonstrates the longest documented follow-up (122 months) among surgically resected PIMT cases with 100% DFS. Nevertheless, some studies report that recurrence may occur even after surgery [11, 33]. Therefore, patients should undergo close surveillance following resection to detect local or distant recurrence. Notably, survival analysis was not performed in our study as all patients remained alive with no recurrence or metastasis.

The histological presentation of PIMT exhibits considerable heterogeneity. A relatively consistent feature is the presence of proliferating myofibroblasts accompanied by varying degrees of mononuclear inflammatory cell infiltration [22]. The inflammatory infiltrate is diverse, frequently composed of lymphocytes and plasma cells, with occasional eosinophils [34]. The density of this infiltrate may obscure the underlying myofibroblasts. Notably, the myofibroblast, the core cellular component of PIMT, represents a distinct cell type intermediate between smooth muscle cells and fibroblasts. Its unique characteristics often render definitive diagnosis challenging even on intraoperative frozen section examination, underscoring the critical importance of immunohistochemical staining for final confirmation [35]. Immunophenotypically, PIMT characteristically expresses mesenchymal markers. In our study, cells demonstrated strong immunoreactivity for SMA in 85.7% of cases, aligning with the general observation of SMA positivity in approximately 80–90% of cases in another reserach [36]. ALK protein expression is also positive in approximately 50–75% of cases [38]with ten cases (71.4%) of patients in our study showing ALK positivity. Additionally, some literature suggests that a Ki67 proliferation index exceeding 25% signifies substantially increased tumor aggressiveness [37]. Notably, all Ki67-positive patients exhibited a Ki67 index ≤ 15% (range, 5–15%) in our cohort. This low proliferative activity may contribute to the absence of recurrence in our cohort. However, histological classification appears to have limited prognostic value, as all patients after R0 resection achieved favorable outcomes regardless of their histopathological subtype.

When surgical resection is not feasible, alternative treatments including radiotherapy, chemotherapy and systemic corticosteroids—may serve as substitutes [34, 38]. ALK tyrosine kinase inhibitors (TKIs), represented by crizotinib, hold significant promise for patients with locally unresectable, advanced, or metastatic ALK-positive IMT [39]. Long-term follow-up indicates a median progression-free survival (PFS) of 18 months and the OS rate of 83.3% in ALK-rearranged patients treated with crizotinib [40]. Although these studies encompassed IMTs from all anatomical sites, the high prevalence of ALK gene rearrangements in PIMT patients similarly supports confidence in the potential benefit of ALK-positive TKIs for PIMT.

While immunotherapy has demonstrated substantial potential in treating numerous malignancies, reports on using immune checkpoint inhibitors for IMT patients are limited. In an analysis of 35 IMT samples by Cottrell et al. [41]28 samples (80%) exhibited PD-L1 positivity in immune cells. Among 20 recurrent and metastatic tumor samples, 16 (80%) were PD-L1 positive. Of eight ALK-negative tumor samples, seven(88%) showed PD-L1 positivity. However, it is critical to note that PD-L1 testing was not performed in our cohort since all patients underwent R0 resection and PD-L1 testing is not routinely indicated for resected PIMT. This PD-L1 positivity suggests that immunotherapy may have therapeutic potential in PIMT. Nevertheless, given the limited availability of clinical reports and the lack of approved indications, it is imperative to conduct prospective trials in the future to validate the effect of PD-L1 positivity for PIMT, especially for those of ALK-negative or unresectable patients.

Though the significance of our study lies in providing crucial clinical insights and favorable prognostic data for this rare disease, the key limitations include its retrospective single-center design and small sample size, inherent to rare disease research. The absence of pediatric cases limits generalizability. Therefore, additional clinical data are required to identify clinical and prognostic features of PIMT.

To the best of our knowledge, this study presents one of the largest single-center surgical series of PIMT cured by surgery; however, our understanding of PIMT remains significantly limited. Due to the lack of specific diagnostic tools, current diagnosis relies on pathology. Surgical resection remains the preferred and effective treatment modality for PIMT, typically yielding satisfactory outcomes. Targeted therapy demonstrates considerable promise, particularly for advanced and ALK-positive PIMT. Given the risk of recurrence and metastasis in some cases, we recommend close, long-term follow-up. Future studies with larger cohorts are warranted to enhance our understanding of PIMT.

## Data Availability

The datasets generated and/or analysed during the current study are not publicly available due to patient privacy.
